# Impact of premature coronary artery disease on adverse event risk following *first* percutaneous coronary intervention

**DOI:** 10.3389/fcvm.2023.1160201

**Published:** 2023-09-07

**Authors:** Tineke H. Pinxterhuis, Eline H. Ploumen, Paolo Zocca, Carine J. M. Doggen, Carl E. Schotborgh, Rutger L. Anthonio, Ariel Roguin, Peter W. Danse, Edouard Benit, Adel Aminian, Marc Hartmann, Gerard C. M. Linssen, Clemens von Birgelen

**Affiliations:** ^1^ Department of Cardiology, Thoraxcentrum Twente, Medisch Spectrum Twente, Enschede, Netherlands; ^2^ Department of Health Technology and Services Research, Faculty BMS, Technical Medical Centre, University of Twente, Enschede, Netherlands; ^3^ Department of Cardiology, Haga Hospital, The Hague, Netherlands; ^4^ Department of Cardiology, Treant Zorggroep, Scheper Hospital, Emmen, Netherlands; ^5^ Department of Cardiology, Hillel Yaffe Medical Center, Hadera and B. Rappaport-Faculty of Medicine, Israel, Institute of Technology, Haifa, Israel; ^6^ Department of Cardiology, Rijnstate Hospital, Arnhem, Netherlands; ^7^ Department of Cardiology, Jessa Hospital, Hasselt, Belgium; ^8^ Department of Cardiology, Centre Hospitalier Universitaire de Charleroi, Charleroi, Belgium; ^9^ Department of Cardiology, Ziekenhuisgroep Twente, Almelo, Hengelo, Netherlands

**Keywords:** coronary artery disease, drug-eluting stent (DES), percutaneous coronary intervention (or PCI), premature coronary artery disease, obstructive coronary artery disease

## Abstract

**Objectives:**

We assessed differences in risk profile and 3-year outcome between patients undergoing percutaneous coronary intervention (PCI) for *premature* and *non-premature* coronary artery disease (CAD).

**Background:**

The prevalence of CAD increases with age, yet some individuals develop obstructive CAD at younger age.

**Methods:**

Among participants in four randomized all-comers PCI trials, without previous coronary revascularization or myocardial infarction (MI), we compared patients with premature (men <50 years; women <55 years) and non-premature CAD. Various clinical endpoints were assessed, including multivariate analyses.

**Results:**

Of 6,171 patients, 887 (14.4%) suffered from premature CAD. These patients had fewer risk factors than patients with non-premature CAD, but were more often smokers (60.7% vs. 26.4%) and overweight (76.2% vs. 69.8%). In addition, premature CAD patients presented more often with ST-segment elevation MI and underwent less often treatment of multiple vessels, and calcified or bifurcated lesions. Furthermore, premature CAD patients had a lower all-cause mortality risk (adj.HR: 0.23, 95%-CI: 0.10–0.52; *p* < 0.001), but target vessel revascularization (adj.HR: 1.63, 95%-CI: 1.18–2.26; *p* = 0.003) and definite stent thrombosis risks (adj.HR: 2.24, 95%-CI: 1.06–4.72; *p* = 0.034) were higher. MACE rates showed no statistically significant difference (6.6% vs. 9.4%; adj.HR: 0.86, 95%-CI: 0.65–1.16; *p* = 0.33)

**Conclusions:**

About one out of seven PCI patients was treated for premature CAD. These patients had less complex risk profiles than patients with non-premature CAD; yet, their risk of repeated revascularization and stent thrombosis was higher. As lifetime event risk of patients with premature CAD is known to be particularly high, further efforts should be made to improve modifiable risk factors such as smoking and overweight.

**TWENTE trials:**

(TWENTE I, clinicaltrials.gov: *NCT01066650*), DUTCH PEERS (TWENTE II, *NCT01331707*), BIO-RESORT (TWENTE III, *NCT01674803*), and BIONYX (TWENTE IV, *NCT02508714*).

## Graphical Abstract

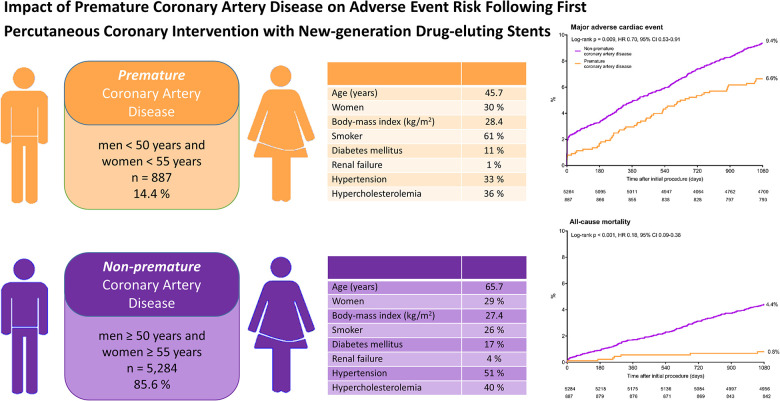
 Impact of premature coronary artery disease on adverse event risk following percutaneous coronary intervention with new-generation drug-eluting stents. Kaplan-Meier cumulative event curves for major adverse cardiac events and all-cause mortality of patients with premature (orange) and *non-*premature (purple) coronary artery disease. CI, confidence interval; HR, hazard ratio.

## 1. Introduction

While coronary artery disease (CAD) has a high prevalence in elderly individuals, for some patients the disease already starts at a young age. Only a small percentage of young individuals develops a stage of CAD that requires coronary revascularization. In individuals aged 40–59 years, the American Heart Association has reported the prevalence of coronary heart disease to be somewhat less than 7% (1). The definition of premature CAD has been changing over time. Earlier studies in adult patients classified CAD as premature, if patients were younger than 45 (or even less than 35) years (2–9), while more recent studies used higher age-thresholds of 50 or 55 years in men (10, 11). As the female hormonal state before menopause delays the progression of atherosclerosis, it makes sense to use a somewhat higher age-threshold in women (12).

Premature atherosclerosis has previously been related to risk factors such a smoking, diabetes, dyslipidemia, and abuse of drugs, such as cocaine or cannabis (2, 7, 9). During the last decades, the cardiovascular risk profile in young individuals has shown subtle alterations. Overall, the prevalence of smoking decreased, and in women the prevalence of hypertension and diabetes increased (13). At the same time, young patients with chronic or acute coronary syndromes do not always have traditional cardiovascular risk factors (14). Awareness of the modifiable risk factors in patients with premature CAD can help set the target for prevention campaigns, in order to reduce the risk of CAD in young individuals. Yet, recent data on the risk profile of all-comers with premature CAD, who underwent percutaneous coronary intervention (PCI) in the current era of new-generation drug-eluting stents (DES) and refined concomitant pharmacological therapy, is scarce.

Therefore, in this pooled patient-level analysis of data from four large-scale randomized PCI trials with contemporary DES, we aimed to assess potential differences in risk profile between patients with premature (men <50 and women <55 years) and non-premature (men ≥50 and women ≥55 years) CAD at the time of their first coronary revascularization procedure. In addition, we assessed and compared the 3-year clinical outcome of both patient groups.

## 2. Methods

### 2.1. Study design

For this study, original patient-level data of all-comer patients enrolled in the TWENTE (clinicaltrials.gov: *NCT01066650*), DUTCH PEERS (TWENTE II, *NCT01331707*), BIO-RESORT (TWENTE III, *NCT01674803*), and BIONYX (TWENTE IV, *NCT02508714*) trials were analyzed. Details of the original trials have been reported previously (15–18). Briefly, these investigator-initiated, patient-blinded, randomized stent trials included 9,204 all-comer patients who underwent a PCI. Trial participants were enrolled in 6 Dutch centers, 2 Belgian centers, and 1 Israeli center. Patients were eligible for trial enrolment if they were older than 18 years, able to give informed consent, and required a PCI with DES implantation. Randomization between stent types was performed in a 1:1 fashion in the TWENTE, DUTCH PEERS, and BIONYX trials, whereas the 3-arm BIO-RESORT trial had a 1:1:1 randomization between 3 stent types. Further details of the four trials are described in the Supplementary Material. A custom-designed computer program with random block sizes of 4 and 8 was used for web-based randomizations. BIO-RESORT and BIONYX stratified for diabetes, while TWENTE and BIONYX stratified for sex. All original trials were approved by the Medical Ethics Committee Twente and the Institutional Review Boards of all participating centers and complied with the Declaration of Helsinki. Written informed consent was provided by all trial participants.

In the present analysis, we compared the cardiovascular risk factors and clinical outcomes between patients with premature CAD and patients with non-premature CAD in those without previous revascularization procedure (by means of PCI or coronary artery bypass surgery) and without previous myocardial infarction (MI). Men were classified as patients with premature CAD if they underwent the index PCI at an age <50 years and likewise women at an age <55 years. Information on cardiovascular risk factors and comorbidities was collected at the time of the index PCI from the medical record or via questionnaires. For all patients, we pooled demographical, clinical, and angiographic characteristics, and outcome data from the four original randomized clinical trials.

### 2.2. Procedures, follow-up, and monitoring

PCI procedures were performed according to international medical guidelines and the operator's judgment. Technical details of the implanted contemporary DES have been published (15–18). In general, dual antiplatelet therapy was prescribed for 12 months in case of an acute coronary syndrome and for 6 months following PCI for a chronic coronary syndrome. Clinical follow-up was obtained via questionnaires, patient visits to outpatient clinics, or by telephone follow-up. The trials were monitored, and adverse events were externally adjudicated by independent blinded clinical event committees.

### 2.3. Definitions

Clinical endpoints were prespecified according to recommendations of the Academic Research Consortium (19, 20). Various clinical endpoints were assessed, including the composite endpoints major adverse cardiac events (MACE; all-cause mortality, any MI, emergent coronary bypass surgery, or clinically indicated target lesion revascularization), target vessel failure (cardiac mortality, target vessel MI, or clinically indicated target vessel revascularization), and target lesion failure (cardiac mortality, any MI, or clinically indicated target lesion revascularization). In addition to each individual endpoint of the composite clinical endpoints, definite and definite-or-probable stent thrombosis were assessed.

### 2.4. Statistical analysis

Chi-square test was used to assess differences in categorical variables, and differences in continuous variables were assessed with the Wilcoxon Rank Sum test or Student *t*-test, as appropriate. Time to endpoints was assessed with the Kaplan-Meier statistics, and the log-rank test was applied for between-group comparisons. Hazard ratios (HR) were computed by Cox proportional hazards analysis, and 2-sided confidence intervals were calculated. Potential confounders were identified if in univariate analysis a *p*-value of <0.15 was found. All potential confounders were included in the first pass of a multivariate Cox regression model. Potential confounders were: diabetes mellitus; body mass index; smoking; renal insufficiency; hypertension; hypercholesterolemia; previous stroke; peripheral arterial disease; family history of coronary artery disease; clinical syndrome at presentation; multivessel treatment; calcified lesion treatment; bifurcated lesion treatment; total stent length and included trial. Stepwise backward selection was used to exclude variables with a non-significant association with the main endpoint. The model consisted of: diabetes mellitus; body mass index; smoking; renal insufficiency; hypertension; family history of coronary artery disease; bifurcated lesion treatment; and total length stent. Finally, a multivariate Cox regression model was used to adjust for the propensity score. Separate, analyses of the events between 1- and 2-year and between 2- and 3-year follow-up were performed. In addition, men and women were divided in 5 percentiles and afterwards one-way analyses of variance were performed to assess between-group difference. *P*-values and confidence intervals were two-sided and *p*-values <0.05 were considered significant. SPSS version 24.00 (IBM, Armonk, NY) was used for the statistical analyses.

## 3. Results

### 3.1. Study population

**Figure 1:**
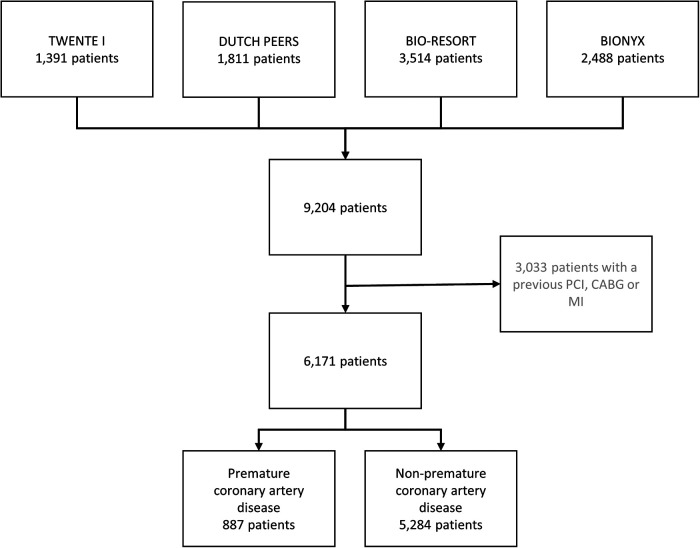
Flowchart. The number of patients with premature coronary artery disease. CABG, coronary artery bypass surgery; MI, myocardial infarction; PCI, percutaneous coronary intervention.

Of all participants in the four randomized clinical trials (*n* = 9,204), 3,033 had a previous coronary revascularization (bypass surgery in 764 patients, and PCI in 1,803 patients) or MI (*n* = 1,896) and were therefore excluded from the present analysis. The current study population consists of 6,171 patients who had no previous coronary revascularization or MI. A total of 887 (14.4%) of these patients had premature CAD (Figure 1), including 269 (30.3%) women <55 years and 618 (69.7%) men <50 years.

### 3.2. Characteristics of patients and procedures

The mean age of patients with premature CAD was 45.7 ± 4.7 years, whereas patients with non-premature CAD were on average 65.7 ± 8.8 years (Table 1). Patients with premature CAD had a lower prevalence of diabetes mellitus, renal failure, hypertension, hypercholesterolemia, peripheral arterial disease, and previous stroke than patients with non-premature CAD. In addition, patients with premature CAD had more often a family history of CAD (59.8% vs. 44.5%), were more often smokers (60.7% vs. 26.4%) and overweight (76.2% vs. 69.8%; body-mass index >25 kg/m^2^. The clinical syndrome at presentation differed between groups: patients with premature CAD presented more often with an ST-segment elevation myocardial infarction (STEMI), while patients with non-premature CAD more frequently presented with stable angina pectoris. This may explain the higher rate of ticagrelor-based dual antiplatelet therapy in patients with premature CAD as compared to patients with non-premature CAD (39.3% vs. 32.6%). In addition, patients with premature CAD were less often treated for multivessel, bifurcated, or calcified coronary lesions, requiring a total stent length that was shorter than in patients with non-premature CAD (Table 1).

**Table 1:** Baseline and procedural characteristics.

	Premature coronary artery disease		*p*-value	
	Yes (*n* = 887)	No (*n* = 5,284)		
General characteristics				
Age (years)	45.7 ± 4.7	65.7 ± 8.8		
Women	269 (30.3)	1,515 (28.7)	0.31	=
Body-mass index (kg/m^2^)	28.4 ± 4.6	27.4 ± 4.2	*<0.001*	↑
Body-mass index (>25 kg/m^2^)	607/797 (76.2)	3,410/4,883 (69.8)	*<0.001*	↑
Smoker	535/881 (60.7)	1,375/5,192 (26.4)	*<0.001*	↑
Medical history				
Diabetes mellitus	94 (10.6)	875 (16.6)	*<0.001*	↓
Renal failure^a^	11 (1.2)	198 (3.7)	*<0.001*	↓
Hypertension	289 (32.7)	2,666 (50.7)	*<0.001*	↓
Hypercholesterolemia	316/881 (35.9)	2,112/5,235 (40.3)	*0.012*	↓
Previous stroke	18 (2.0)	267 (5.1)	*<0.001*	↓
Peripheral arterial disease^b^	18 (2.0)	346 (6.6)	*<0.001*	↓
LVEF < 30%	5 (0.6)	59 (1.2)	0.13	↓
Family history of coronary artery disease	523/875 (59.8)	2,294/5,153 (44.5)	<0.001	↑
Clinical syndrome at presentation			*<0.001*	
Stable angina pectoris	177 (20.0)	1,696 (32.1)		
STEMI	358 (40.4)	1,464 (27.7)		
Non-STEMI	224 (25.3)	1,277 (24.2)		
Unstable angina pectoris	128 (14.4)	847 (16.0)		
DAPT			*0.003*	
Clopidogrel-based DAPT	320/528 (60.7)	2,036/3,029 (67.4)		
Ticagrelor-based DAPT	207/528 (39.3)	987/3,029 (32.6)		
Procedural characteristics				
Multivessel treatment	117 (13.2)	984 (18.6)	*<0.001*	↓
Target vessel				
Left main stem	12 (1.4)	63 (1.2)	0.69	=
Right coronary artery	321 (36.2)	1,936 (36.6)	0.80	=
Left anterior descending artery	472 (53.2)	2,838 (53.7)	0.78	=
Left circumflex artery	207 (23.3)	1,514 (28.7)	*<0.001*	↓
Total stent length (mm)	34.8 ± 23.5	38.4 ± 26.1	*<0.001*	↓
Calcified lesion treatment	91 (10.3)	1,070 (20.2)	*<0.001*	↓
Ostial lesion treatment	37 (4.2)	286 (5.4)	0.13	=
Bifurcated lesion treatment^c^	263 (29.7)	1,841 (34.8)	*0* *.* *003*	↓
Chronic total occlusion treatment	36 (4.1)	224 (4.2)	0.80	=

Values are mean ± SD, *n* (%) or *n*/*N* (%). Procedures present patient-level data. DAPT, dual antiplatelet therapy; LVEF, Left ventricle ejection fraction; Non-STEMI, non–ST-segment–elevation myocardial infarction; STEMI, ST-segment–elevation myocardial infarction.

^a^ Defined as previous renal failure, creatinine ≥130 μmol/L, or the need for dialysis.

^b^ Defined as a history of: symptomatic atherosclerotic lesion in the lower or upper extremities; atherosclerotic lesion in the aorta causing symptoms or requiring treatment; atherosclerotic lesion in the carotid or vertebral arteries related to a non-embolic ischemic cerebrovascular event; or symptomatic atherosclerotic lesion in a mesenteric artery.

^c^ Target lesions were classified as bifurcated if a side branch ≥1.5 mm originated from them.

Italic values are statically significant.

### 3.3. Clinical outcome at 3-year follow-up

**Figure 2:**
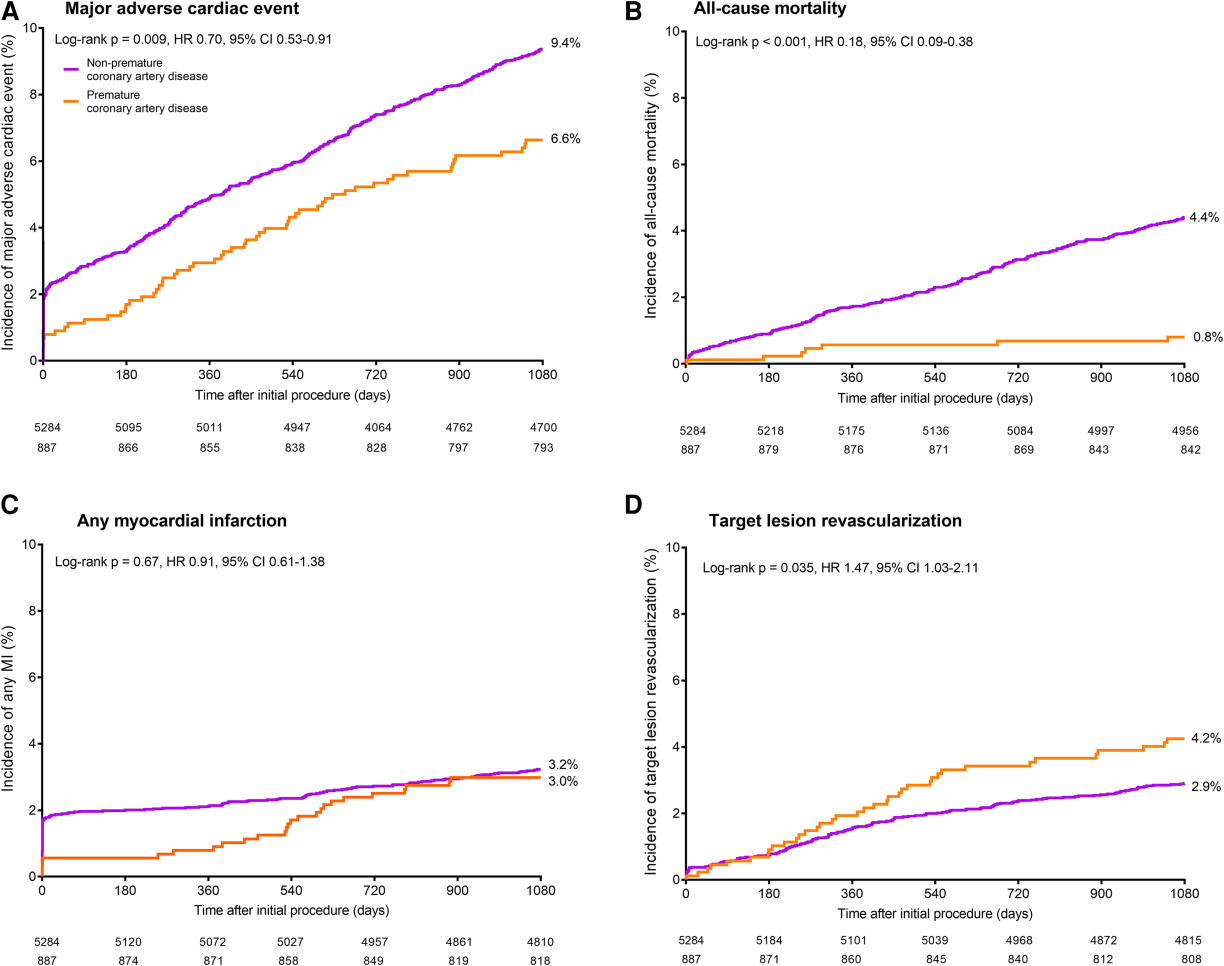
Kaplan–Meier cumulative event curves for the endpoint major adverse cardiac events and its individual components at 3-year follow-up. Kaplan–Meier cumulative incidence curves for: (**A**) the primary endpoint major adverse cardiac events, a composite of all-cause mortality (**B**), any myocardial infarction (**C**), emergent coronary bypass surgery, or clinically indicated target lesion revascularization (**D**) Patients with premature (orange) and *non-*premature (purple) coronary artery disease. HR, hazard ratio; MI, myocardial infarction.

During 3-year follow-up, the composite clinical endpoint MACE occurred in 58/887 (6.6%) patients with premature CAD and in 492/5,284 (9.4%) patients with non-premature CAD (*p* = 0.009; Figure 2). In addition, patients with premature CAD had lower rates of all-cause mortality (HR: 0.18, 95%-CI: 0.09–0.38; *p* < 0.001) and cardiac mortality (HR: 0.26, 95%-CI: 0.10–0.70; *p* = 0.004) than patients with non-premature CAD. In contrast, patients with premature CAD had higher rates of definite stent thrombosis (HR: 2.14, 95%-CI: 1.00–4.58; *p* = 0.045), target vessel revascularization (HR: 1.41, 95%-CI: 1.05–1.90; *p* = 0.024), and target lesion revascularization (HR: 1.47, 95%-CI: 1.03–2.11; *p* = 0.035; Table 2). Very late (>1 year) definite stent thrombosis showed no statistically significant difference between both groups (5/887 vs. 12/5,284 patients; *p* = 0.08). Inspection of the Kaplan-Meier time-to-event curves suggested potential between-group differences for MI and target vessel revascularization during follow-up, which triggered the calculation of annual adverse event rates that are presented online in the Supplementary Material.

**Table 2:** Clinical outcomes at 3-year.

	Premature coronary artery disease		HR (95%-CI)	*p*_log-rank_	Forrest plot	Adjusted HR^a^ (95-CI)	*p*-value
	Yes (*n* = 887)	No (*n* = 5,284)					
All-cause mortality	7 (0.8)	231 (4.4)	0.18 (0.09–0.38)	*<0.001*	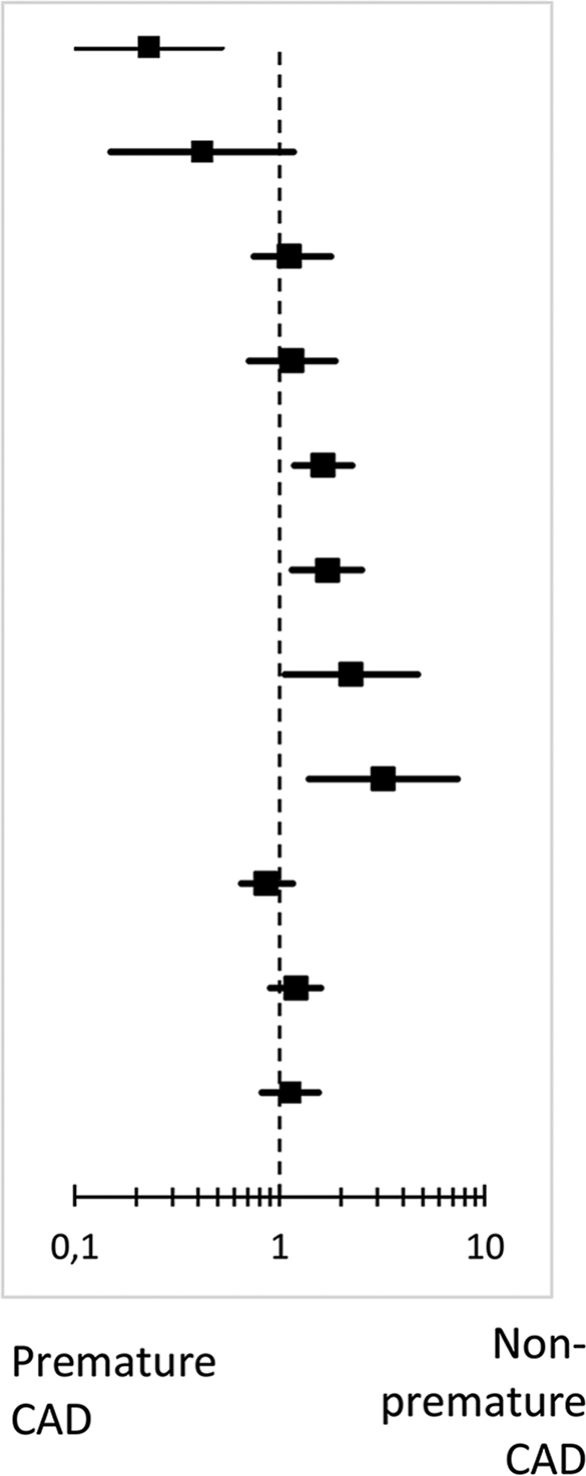	0.23 (0.10–0.52)	*<0.001*
Cardiac mortality	4 (0.5)	92 (1.8)	0.26 (0.10–0.70)	*0.004*		0.42 (0.15–1.17)	0.10
Any myocardial infarction	26 (3.0)	168 (3.2)	0.91 (0.61–1.38)	0.67		1.12 (0.75–1.78)	0.52
Target vessel related MI	20 (2.3)	139 (2.7)	0.85 (0.53–1.36)	0.50		1.15 (0.71–1.87)	0.58
Target vessel revascularization	53 (6.1)	224 (4.3)	1.41 (1.05–1.90)	*0* *.* *024*		1.63 (1.18–2.26)	*0* *.* *003*
Target lesion revascularization	37 (4.2)	150 (2.9)	1.47 (1.03–2.11)	*0.035*		1.71 (1.15–2.52)	*0.008*
Definite-or-probable stent thrombosis	10 (1.1)	37 (0.7)	1.60 (0.80–3.23)	0.18		2.24 (1.06–4.72)	*0.034*
Definite stent thrombosis	9 (1.0)	25 (0.5)	2.14 (1.00–4.58)	*0.045*		3.20 (1.39–7.38)	*0* *.* *006*
Major adverse cardiac events^b^	58 (6.6)	492 (9.4)	0.70 (0.53–0.91)	*0.009*		0.86 (0.65–1.16)	0.33
Target vessel failure^c^	64 (7.3)	399 (7.6)	0.95 (0.73–1.24)	0.69		1.20 (0.90–1.59)	0.21
Target lesion failure^d^	50 (5.7)	338 (6.5)	0.87 (0.65–1.18)	0.38		1.13 (0.82–1.55)	0.46

Data are *n* (%), unless otherwise indicated. MI, myocardial infarction.

^a^ Propensity score including the possible confounders: diabetes mellitus; smoking, renal insufficiency; previous coronary artery bypass surgery; previous MI; clinical diagnosis at presentation; ostial lesion treatment; and total length stent.

^b^ Major adverse cardiac events is a composite of all-cause mortality, any myocardial infarction, emergent coronary artery bypass surgery, and clinically indicated target lesion revascularization.

^c^ Target vessel failure is a composite of cardiac mortality, target vessel related myocardial infarction, and clinically indicated target vessel revascularization.

^d^ Target lesion failure is a composite of cardiac mortality, target vessel related myocardial infarction, and clinically indicated target lesion revascularization.

Italic values are statically significant.

After adjustment for confounders, the risk for all-cause mortality was significantly lower in patients with premature CAD (adj.HR: 0.23, 95%-CI: 0.10–0.52; *p* < 0.001), while in these patients the risks of target vessel revascularization (adj.HR: 1.63, 95%-CI: 1.18–2.26; *p* = 0.003), target lesion revascularization (adj.HR: 1.71, 95%-CI: 1.15–2.52; *p* = 0.008), definite-or-probable stent thrombosis (adj.HR: 2.24, 95%-CI: 1.06–4.72; *p* = 0.034), and definite stent thrombosis (adj.HR: 3.20, 95%-CI: 1.39–7.38; *p* = 0.006) were significantly higher than in patients with non-premature CAD.

When dividing men and women into 5 age percentiles, the rates of MACE, and all-cause and cardiac mortality were found to be lower in younger as compared to older men and women (Table 3). Similar results were found when men and women were divided in 7-age groups (Supplementary Table S2).

**Table 3:** Clinical outcomes in men and women at 3-year follow-up divided in 5 percentiles based on age.

Men	Five age groups				
	<53 years	53–59 years	60–64 years	65–71 years	>72 years
	*n* = 877	*n* = 878	*n* = 877	*n* = 878	*n* = 877
All-cause mortality	5 (0.6)	11 (1.3)	25 (2.9)	30 (3.4)*	77 (8.8)*
Cardiac mortality	4 (0.5)	3 (0.3)	13 (1.5)	7 (0.8)	30 (3.4)*
Any myocardial infarction	29 (3.3)	22 (2.5)	31 (3.5)	27 (3.1)	28 (3.2)
Target vessel related MI	20 (2.3)	17 (1.9)	25 (2.9)	25 (2.8)	23 (2.6)
Target vessel revascularization	53 (6.0)	42 (4.8)	38 (4.3)	45 (5.1)	31 (3.5)
Target lesion revascularization	36 (4.1)	29 (3.3)	30 (3.4)	25 (2.8)	19 (2.2)
Definite-or-probable stent thrombosis	10 (1.1)	4 (0.5)	8 (0.9)	8 (0.9)	5 (0.6)
Definite stent thrombosis	9 (1.0)	2 (0.2)	5 (0.6)	7 (0.8)	3 (0.3)
Major adverse cardiac events^a^	58 (6.6)	57 (6.5)	74 (8.4)	68 (7.7)	110 (12.5)*
Target vessel failure^b^	64 (7.3)	56 (6.4)	65 (7.4)	59 (6.7)	71 (8.1)
Target lesion failure^c^	49 (5.6)	46 (5.2)	59 (6.7)	44 (5.0)	60 (6.8)
Women	<58 years	58–64 years	65–70 years	71–76 years	>76 years
	*n* = 357	*n* = 357	*n* = 357	*n* = 357	*n* = 356
All-cause mortality	4 (1.1)	9 (2.5)	15 (4.2)	25 (7.0)*	37 (10.4)*
Cardiac mortality	1 (0.3)	2 (0.6)	5 (1.4)	14 (3.9)*	17 (4.8)*
Any myocardial infarction	6 (1.7)	13 (3.6)	12 (3.4)	18 (5.0)	8 (2.2)
Target vessel related MI	5 (1.4)	12 (3.4)	8 (2.2)	16 (4.5)	8 (2.2)
Target vessel revascularization	17 (4.8)	15 (4.2)	14 (3.9)	16 (4.5)	6 (1.7)
Target lesion revascularization	12 (3.4)	13 (3.6)	11 (3.1)	6 (1.7)	6 (1.7)
Definite-or-probable stent thrombosis	2 (0.6)	2 (0.6)	1 (0.3)	4 (1.1)	3 (0.8)
Definite stent thrombosis	2 (0.6)	1 (0.3)	1 (0.3)	3 (0.8)	1 (0.4)
Major adverse cardiac events^a^	21 (5.9)	33 (9.2)	37 (10.4)	44 (12.3)	48 (13.5)*
Target vessel failure^b^	22 (6.2)	27 (7.6)	27 (7.6)	43 (12.0)	29 (8.1)
Target lesion failure^c^	17 (4.8)	25 (7.0)	24 (6.7)	35 (9.8)	29 (8.1)

Data are *n* (%), unless otherwise indicated. MI, myocardial infarction.

^a^ Major adverse cardiac events is a composite of all-cause mortality, any myocardial infarction, emergent coronary artery bypass surgery, and clinically indicated target lesion revascularization.

^b^ Target vessel failure is a composite of cardiac mortality, target vessel related myocardial infarction, and clinically indicated target vessel revascularization.

^c^ Target lesion failure is a composite of cardiac mortality, target vessel related myocardial infarction, and clinically indicated target lesion revascularization.

* *p*-value of <0.05.

## 4. Discussion

### 4.1. Main findings

Patients with premature CAD had less often diabetes mellitus, renal failure, hypertension, hypercholesterolemia, and a history of stroke; yet, they had on average a higher body-mass index and were more often smokers. Furthermore, patients with premature CAD underwent less often treatment of multiple vessels and of calcified or bifurcated coronary lesions. At 3-year follow-up, the rate of MACE differed between patients with premature and non-premature CAD (6.6% vs. 9.4%), but the presence of premature coronary atherosclerosis was not an independent predictor of MACE. In addition, 3-year all-cause mortality was lower in patients with premature CAD, while the rates of repeated revascularization and stent thrombosis were higher. When men and women were divided into age percentiles, the rates of MACE, and all-cause and cardiac mortality were found to be lower in younger patients.

### 4.2. Definition of premature coronary artery disease

The definition of “premature CAD” (or “premature atherosclerosis” or “young adults with an MI”) varies between studies and several different age-thresholds have been used. In most recent studies, age-thresholds of 45, 50, or 55 years were used for premature atherosclerosis (10, 11, 21). Yet, some studies applied a lower age-threshold of 35 or 40 years (6, 22), while other researchers divided their patients in 3 groups: very young, young, and older (23, 24). A study based on the autopsy reports of 1,139 men with sudden coronary atherosclerotic death, recommended an age-threshold of 49 years for the definition of premature CAD (25). Yet, as the female hormonal state delays the progression of atherosclerosis, women generally develop atherosclerosis at a more advanced age. Therefore, it makes sense to apply somewhat different age-thresholds in men and women. A previous study also used age-thresholds of 50 years in men and 55 years in women to define premature atherosclerosis (26). The definition of premature CAD in our present manuscript is in line with that definition.

### 4.3. Prevalence of premature coronary artery disease

From 2015 to 2018, the American Heart Association reported the prevalence of coronary heart disease in men and women aged 40–59 years to be 6.9% and 6.6% (1). The prevalence of premature CAD differs between studies; it is about 20%–30% in patients presenting with a MI (10, 13). It may differ between the various regions of the world and among ethnicities. For instance, an Asian study of patients with premature CAD performed in India found a prevalence of 26.5% (27). In mostly European patients without previous revascularization or MI, assessed in the present study, this rate was 14.4%. Over the years, a stabilization or even an increase in the number of young adults with obstructive CAD have been observed (1, 10, 13), which may be explained by an increase in cardiac risk profile and relevant comorbidities (28), which emphasizes the importance of assessing modifiable cardiovascular risk factors and optimizing their treatment in young adults.

### 4.4. Risk factors

In the present analysis, patients with premature CAD had significantly less often hypertension, hypercholesterolemia, and diabetes, which is in line with previous studies (6, 8, 22, 29–31). In addition, our patients with premature CAD had on average a higher body-mass index, presented more often with STEMI, and underwent less often multivessel treatment than patients with non-premature CAD. Furthermore, patients with premature CAD were significantly more often current smokers.

Previous studies found that patients with premature CAD, who required PCI, were more likely to be men, as compared to patients with non-premature CAD. Yet, patients with premature CAD suffered less often from heart failure, hypertension, diabetes, or a history of coronary revascularization (6, 8, 22, 27, 29–31). In addition, patients with premature CAD had on average a higher body-mass index (6, 7). Furthermore, they presented more often with STEMI, had more often single-vessel disease, and a lower total plaque burden (22, 24, 29, 30). The data on smoking status were contradictory: one study found no difference in the current smoking status (29), while other studies found a higher proportion of current smokers among the premature CAD patients (6, 22, 30, 31).

In addition, three large-scale studies that assessed only young patients with coronary artery disease found a high prevalence of smoking, hypercholesterolemia, obesity, and family history of CAD (32–34). This is consistent with the findings of the present analysis.

### 4.5. Chronological versus biological aging

It is still unclear why some patients develop obstructive CAD at a much younger age than others. Yet, it is likely that a combination of genetic predisposition, for instance the risk for familial hypercholesterolemia, and cardiovascular risk factors accounts for the premature occurrence of CAD (35). Observations have indicated that biological or physiological aging, which relates to a decline in function, differs from chronological aging, which only involves time. Physiological aging involves, for instance: telomere shorting and attrition (36); DNA methylation which is a epigenetic mechanism that regulates gene expression (37); and accumulation of mutations in DNA of somatic cells (38). In addition, a low-grade inflammation, measured by elevated proinflammatory molecules, is also a characteristic of aging (39). Furthermore, changes in the levels of proteins, metabolites, and other biomolecules have been observed (40). With aging, structural changes in arteries such as elastin fragmentation, collagen accumulation, medial vascular smooth muscle cell loss, and atherosclerosis have been reported (41).

In addition, several differences in plaques characteristics have been observed between patients with premature and non-premature CAD, which may be related to differences in the pathophysiological mechanisms involved and may explain the differences in clinical outcome. In the present study, plaque characteristics were not assessed in a detailed way. Yet, other studies observed that young adults have fewer atherosclerotic lesions and relative lack of acellular scar tissue, but their plaques have more inflammatory features and contain more lipid-containing foam cells (2, 7). Furthermore, young adults with STEMI showed a higher prevalence of plaque erosion and a lower degree of luminal obstruction, while other plaque features (e.g., calcification, cholesterol crystals, and thin-cap fibroatheroma) were less often seen (31).

### 4.6. Clinical outcome and implications

The present study found in patients with premature CAD a lower 3-year all-cause mortality risk than in patients with non-premature CAD. Yet, premature CAD patients had higher risks of target vessel revascularization, target lesion revascularization, and definite stent thrombosis. The number of patients with a repeated revascularization may be higher due to the fact that generally, young patients are more physically active and therefore more likely to develop symptoms of angina. In addition, in young adults with symptoms, physicians may be more inclined to choose an invasive treatment, such as a PCI, than choosing pharmacological therapy only.

A previous study which assessed patients aged ≤40 years who underwent coronary revascularization found 6-months and 1-year mortality rates that were lower than in non-premature CAD patients (8). This is in line with the results of the current analysis at 3-year follow-up. Similarly, the Norwegian Myocardial Infarction Register found that young MI patients (<45 years) had a 40% lower risk of all-cause mortality after a median follow-up of 2.4 years than MI patients aged between 45 and 59 years (30).

It is important to identify individuals who are at a particularly high risk of secondary adverse events in order to take more aggressive preventive measures. In the present analysis of patients undergoing their first coronary revascularization, a significant proportion had modifiable risk factors, such as hypertension, hypercholesterolemia, overweight, or smoking. As the prevalence of premature CAD increases with the number of cardiovascular risk factors (1, 28), increasing patient awareness about modifiable risk factors will support secondary cardiovascular prevention and is likely to improve the clinical outcome in PCI patients with premature CAD. In addition, rigorous screening for modifiable risk factors and the presence of atherosclerosis (primary prevention) in asymptomatic descendants of patients with premature CAD could be beneficial.

### 4.7. Limitations

Based on the *post hoc* nature of the present analysis, our findings should be considered hypothesis generating. The analysis assessed patients without previous revascularization or previous MI in order to prevent that patients of the non-premature CAD group could have met the criteria of premature CAD previously. While using this criterion was indispensable, it might have resulted in the exclusion of some patients with extensive atherosclerotic burden. In addition, comparison between studies can be difficult due to dissimilarities between the various used definitions of premature CAD and the study populations. As other studies, we cannot exclude the presence of undetected or unmeasured confounders. Data about medication compliance were not available and could therefore not be assessed. The present analysis, similar to previous research, used chronological age instead of biological age, as biological age estimators are more complex and quite impractical in the context of most studies.

## 5. Conclusions

About one out of seven PCI patients was treated for premature CAD. These patients had less complex risk profiles than patients with non-premature CAD; yet, their risk of repeated revascularization and stent thrombosis was higher. As lifetime event risk of patients with premature CAD is known to be particularly high, further efforts should be made to improve modifiable risk factors such as smoking and overweight.

## Data Availability

The datasets presented in this article are not readily available because of the privacy of the participants. Researchers with a specific research question can send it to the corresponding author. The request will be assessed individually by a group of searchers consisting of members of the steering committees of the trials. Requests to access the datasets should be directed to C. von Birgelen, c.vonbirgelen@mst.nl.
