# Supplementary material for: Impact of premature coronary artery disease on adverse event risk following first percutaneous coronary intervention
Source: Front Cardiovasc Med. 2023 Sep 7;10:1160201. doi: 10.3389/fcvm.2023.1160201 (PMC10512829; doi:10.3389/fcvm.2023.1160201)
Supplement: Supplementary file 1 [file Datasheet1.pdf]

Impact of Premature Coronary Artery Disease on Adverse Event Risk Following *First* Percutaneous Coronary Intervention

Tineke H. Pinxterhuis MD[a,b], Eline H. Ploumen MD PhD[a,b], Paolo Zocca MD PhD[a], Carine J.M. Doggen PhD[b], Carl E. Schotborgh MD[c], Rutger L. Anthonio MD PhD[d], Ariel Roguin MD PhD[e], Peter W. Danse MD PhD[f], Edouard Benit MD[g], Adel Aminian MD[h], Marc Hartmann MD PhD[a], Gerard C.M. Linssen MD PhD[i], Clemens von Birgelen MD PhD[a,b]

a. Department of Cardiology, Thoraxcentrum Twente, Medisch Spectrum Twente, Enschede, the Netherlands
b. Department of Health Technology and Services Research, Faculty BMS, Technical Medical Centre, University of Twente, Enschede, the Netherlands
c. Department of Cardiology, Haga Hospital, The Hague, the Netherlands
d. Department of Cardiology, Treant Zorggroep, Scheper Hospital, Emmen, the Netherlands
e. Department of Cardiology, Hillel Yaffe Medical Center, Hadera and B. Rappaport-Faculty of Medicine, Israel, Institute of Technology, Haifa, Israel
f. Department of Cardiology, Rijnstate Hospital, Arnhem, the Netherlands
g. Department of Cardiology, Jessa Hospital, Hasselt, Belgium
h. Department of Cardiology, Centre Hospitalier Universitaire de Charleroi, Charleroi, Belgium
i. Department of Cardiology, Ziekenhuisgroep Twente, Almelo and Hengelo, the Netherlands

SUPPLEMENTAL MATERIAL

Index

Supplemental Methods ______ *page 2*

Annual event rates ______ *page 3*

Supplemental Table 1: Clinical events between 0- and 1-year, 1- and 2-year and 2- and 3-year follow-up ______ *page 4*

Supplemental Table 2: Clinical outcomes in men and women at 3-year follow-up divided in 7 groups based on age ______ *page 8*

Supplemental Methods

The DUTCH PEERS, BIO-RESORT and BIONYX trials enrolled patients who required PCI for any coronary syndrome, while TWENTE trial excluded patients with an ST-segment elevation myocardial infarction. The TWENTE trial included patients who presented in the Thoraxcentrum Twente, Enschede (the Netherlands) and assessed the safety and efficacy of Resolute zotarolimus-eluting stent (Medtronic Inc) and the Xience V everolimus-eluting stent (Abbott Vascular).

The DUTCH PEERS included patients in Thoraxcentrum Twente; Rijnstate Hospital (Arnhem, the Netherlands); Scheper Hospital (Emmen, the Netherlands) and Medisch Centrum Alkmaar (Alkmaar, the Netherlands) and assessed the Resolute Integrity zotarolimus-eluting stent (Medtronic) and Promus Element everolimus-eluting stent (Boston Scientific).

The BIO-RESORT enrolled patients in Thoraxcentrum Twente; Rijnstate Hospital; the Haga Hospital (Den Haag, the Netherlands) and Albert Schweitzer Hospital (Dordrecht, the Netherlands) and assessed in all-comers the safety and efficacy of Orsiro sirolimus-eluting stents (Biotronik) and Synergy everolimus-eluting stents (Boston Scientific) versus Resolute Integrity zotarolimus-eluting stents (Medtronic).

The BIONYX trial included patients in 4 Dutch hospitals (Thoraxcentrum Twente, Enschede; Haga Hospital, Den Haag; Rijnstate Hospital, Arnhem; Scheper Hospital, Emmen), 2 Belgium hospitals (Virga Jessa Hospital, Hassalt; University Hospital Charleroi, Charleroi) and 1 hospital in Israel (Rambam Hospital, Haifa) and compared Orsiro sirolimus-eluting stents (Biotronik) versus Resolute Onyx zotarolimus-eluting stents (Medtronic).

Annual event rates

During the first year of follow-up, patients with premature CAD had lower rates of MACE (HR:0.61, 95%-CI:0.41-0.91; p=0.014), all-cause mortality (HR:0.33, 95%-CI:0.13-0.81; p=0.011), any MI (HR:0.37, 95%-CI:0.17-0.80; p=0.008), and target vessel-related MI (HR:0.40, 95%-CI:0.18-0.85; p=0.013) than patients with non-premature CAD. Between 1- and 2-year follow-up, patients with premature CAD showed lower rates of all-cause mortality (HR:0.08, 95%-CI:0.01-0.57; p<0.001), while –in contrast to the first year of follow-up– they had higher rates of any MI (HR:2.81, 95%-CI:1.52-5.19; p<0.001) and target vessel-related MI (HR:3.78, 95%-CI:1.83-7.78; p<0.001) than in patients with non-premature CAD. In addition, during the second year of follow-up the rates of target lesion revascularization (HR:1.85, 95%-CI:0.99-3.45; p=0.048) and stent thrombosis (HR:2.99, 95%-CI:1.02-8.75; p=0.036) were also higher in patients with premature CAD. The analysis of adverse events that occurred between 2- and 3-year follow-up revealed a lower rate of all-cause mortality (HR:0.09, 95%-CI:0.01-0.66; p=0.003) in patients with premature CAD as compared to patients with non-premature CAD (Table). The results suggest that the higher rates of repeated revascularization and stent thrombosis were mainly driven by events that occurred during the second year of follow-up. During the first year of follow-up, patients with premature CAD experienced less often MI than patients with non-premature CAD

Supplemental Table 1: Clinical events between 0- and 1-year, 1- and 2-year and 2- and 3-year follow-up

	Premature coronary artery disease		HR (95%-CI)	$P_{log-rank}$	Forrest plot
	Yes (n=887)	No (n=5,284)			
	**Events between 0 and 1 year**				
**All-cause mortality**	5 (0.6)	90 (1.7)	0.33 (0.13-0.81)	*0.011*	
**Cardiac mortality**	3 (0.3)	48 (0.9)	0.37 (0.12-1.19)	0.083	
**Any myocardial infarction**	7 (0.8)	111 (2.1)	0.37 (0.17-0.80)	*0.008*	
**Target vessel related MI**	7 (0.8)	105 (2.0)	0.40 (0.18-0.85)	*0.013*	
**Target vessel revascularization**	22 (2.5)	119 (2.3)	1.10 (0.70-1.73)	0.69	
**Target lesion revascularization**	17 (1.9)	80 (1.5)	1.26 (0.75-2.13)	0.38	
**Definite-or-probable stent thrombosis**	5 (0.6)	21 (0.4)	1.42 (0.53-3.76)	0.48	
**Definite stent thrombosis**	4 (0.5)	13 (0.2)	1.83 (0.60-5.61)	0.28	
**Major adverse cardiac events[a]**	27 (2.9)	261 (4.9)	0.61 (0.41-0.91)	*0.014*	
**Target vessel failure[b]**	29 (3.3)	245 (4.7)	0.70 (0.48-1.03)	0.07	
**Target lesion failure[c]**	24 (2.7)	211 (4.0)	0.67 (0.44-1.03)	0.06	

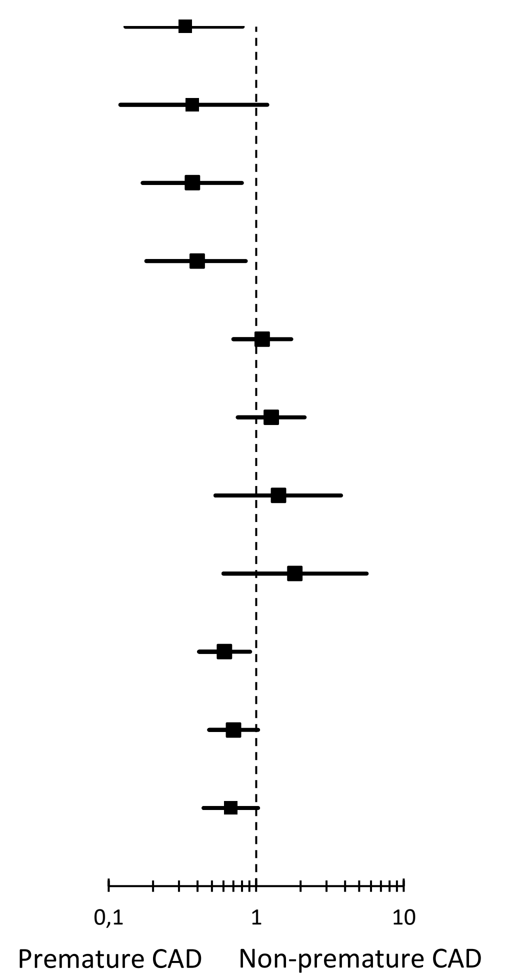

Events between 1 and 2 year				
**All-cause mortality**	1 (0.1)	75 (1.4)	0.08 (0.01-0.57)	*<0.001*
**Cardiac mortality**	0	26 (0.5)		
**Any myocardial infarction**	15 (1.7)	32 (0.6)	2.81 (1.52-5.19)	*<0.001*
**Target vessel related MI**	12 (1.4)	19 (0.4)	3.78 (1.83-7.78)	*<0.001*
**Target vessel revascularization**	19 (2.2)	58 (1.1)	1.96 (1.17-3.30)	0.09
**Target lesion revascularization**	13 (1.5)	42 (0.8)	1.85 (0.99-3.45)	*0.048*
**Definite-or-probable stent thrombosis**	5 (0.6)	10 (0.2)	2.99 (1.02-8.75)	*0.036*
**Definite stent thrombosis**	5 (0.6)	6 (0.1)	4.98 (1.52-16.32)	*0.003*
**Major adverse cardiac events[a]**	21 (2.4)	132 (2.5)	0.95 (0.60-1.51)	0.84
**Target vessel failure[b]**	25 (2.5)	145 (2.5)	1.03 (0.68- 1.58)	0.88
**Target lesion failure[c]**	17 (1.9)	75 (1.4)	1.36 (0.80-2.30)	0.25

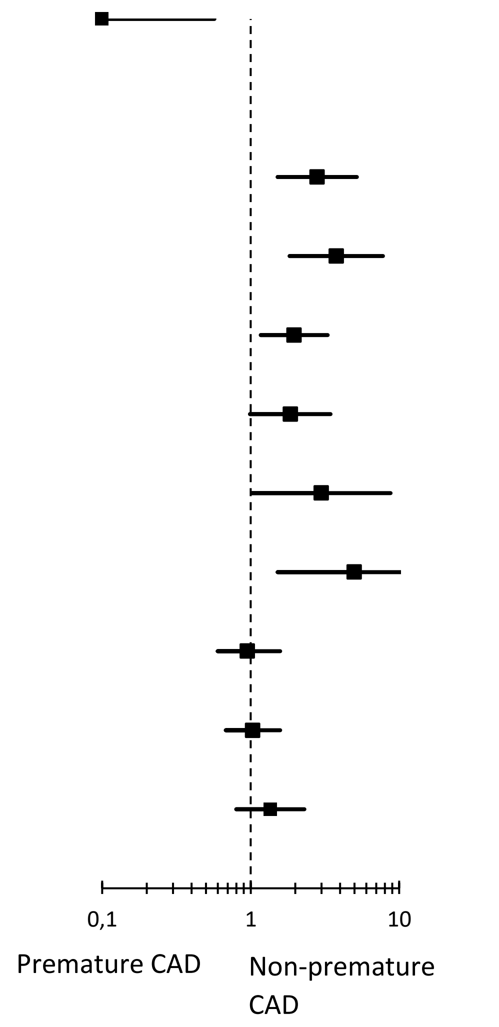

Events between 2 and 3 year				
**All-cause mortality**	1 (0.1)	66 (1.3)	0.09 (0.01-0.66)	*0.003*
**Cardiac mortality**	1 (0.1)	18 (0.4)	0.34 (0.05-2.51)	0.26
**Any myocardial infarction**	4 (0.5)	25 (0.5)	0.97 (0.34-2.77)	0.95
**Target vessel related MI**	1 (0.1)	15 (0.3)	0.40 (0.05-3.04)	0.36
**Target vessel revascularization**	12 (1.4)	47 (0.9)	1.54 (0.82-2.90)	0.18
**Target lesion revascularization**	7 (0.8)	27 (0.5)	1.56 (0.68-3.58)	0.29
**Definite-or-probable stent thrombosis**	0	6 (0.1)		
**Definite stent thrombosis**	0	6 (0.1)		
**Major adverse cardiac events**[a]	11 (1.3)	103 (2.0)	0.64 (0.35-1.20)	0.16
**Target vessel failure**[b]	14 (1.6)	66 (1.3)	1.28 (0.72- 2.28)	0.40
**Target lesion failure**[c]	9 (1.0)	52 (1.0)	1.04 (0.51-2.12)	0.91

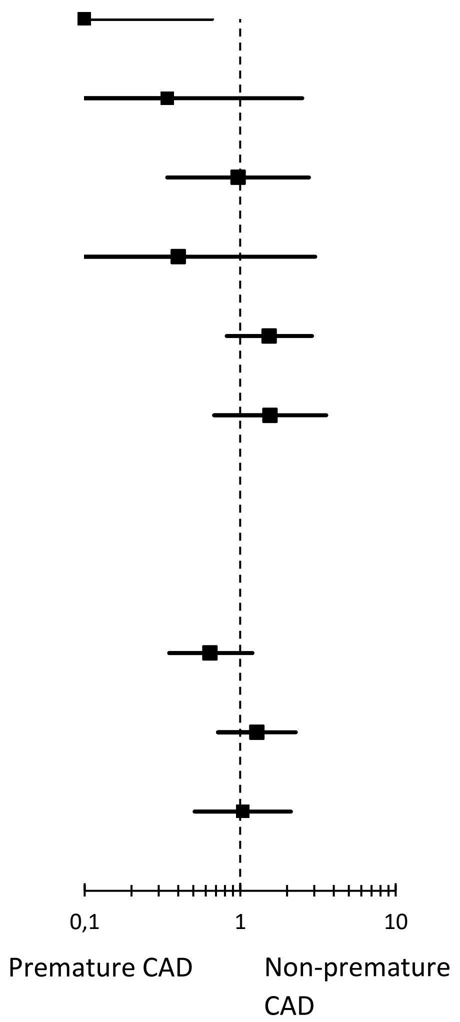

Data are n (%), unless otherwise indicated. [a]Major adverse cardiac events is a composite of all-cause mortality, any myocardial infarction, emergent coronary artery bypass surgery, and clinically indicated target lesion revascularization. [b]Target vessel failure is a composite of cardiac mortality, target vessel related myocardial infarction, and clinically indicated target vessel revascularization. [c]Target lesion failure is a composite of cardiac mortality, target vessel related myocardial infarction, and clinically indicated target lesion revascularization.
Abbreviations: MI = myocardial infarction

Supplemental Table 2: Clinical outcomes in men and women at 3-year follow-up divided in 7 groups based on age

	7 Age groups						
**Men**	<50 years n=618	50-54 years n=561	55-59 years n=716	60-64 years n=742	65-69 years n=743	70-74 years n=503	≥75 years n=504
**All-cause mortality**	4 (0.7)	2 (0.4)	13 (1.8)	22 (3.0)*	24 (3.3)*	30 (6.0)*	53 (10.6)*
**Cardiac mortality**	3 (0.7)	2 (0.4)	4 (0.6)	11 (0.8)	6 (1.5)	12 (2.4)*	19 (3.9)*
**Any myocardial infarction**	21 (3.5)	15 (2.7)	20 (2.8)	26 (3.5)	22 (3.0)	13 (2.6)	20 (4.1)
**Target vessel related MI**	16 (2.6)	11 (2.0)	13 (1.8)	22 (3.0)	20 (2.7)	12 (2.4)	16 (3.2)
**Target vessel revascularization**	40 (6.6)	26 (4.7)	38 (5.4)	29 (4.0)*	38 (5.2)	20 (4.1)	18 (3.7)*
**Target lesion revascularization**	28 (4.6)	18 (3.2)	26 (3.7)	23 (3.1)	22 (3.0)	10 (2.0)	12 (2.5)
**Definite-or-probable stent thrombosis**	8 (1.3)	5 (0.9)	2 (0.3)	7 (1.0)	8 (1.1)	2 (0.4)	3 (0.6)
**Definite stent thrombosis**	7 (1.1)	3 (0.5)	1 (0.1)	5 (0.7)	7 (1.0)	1 (0.2)	2 (0.4)
**Major adverse cardiac events †**	42 (6.9)	33 (5.9)	52 (7.3)	62 (8.4)	56 (7.6)	48 (9.6)	74 (14.7)*
**Target vessel failure ‡**	47 (7.7)	35 (6.2)	50 (7.0)	53 (7.2)	49 (6.7)	39 (7.9)	42 (8.5)
**Target lesion failure §**	37 (6.1)	29 (5.2)	40 (5.6)	48 (6.5)	37 (5.0)	30 (6.0)	37 (7.5)
**Women**	<55 years n=269	55-59 years n=192	60-64 years n=257	65-69 years n=324	70-74 years n=294	75-79 years n=448	≥80 years
**All-cause mortality**	3 (1.1)	2 (1.0)	9 (3.5)	12 (3.7)	15 (5.1)*	24 (9.6)*	25 (13.0)*
**Cardiac mortality**	1 (0.4)	0	2 (0.8)	3 (0.9)	8 (2.8)	13 (5.3)*	12 (6.4)*
**Any myocardial infarction**	5 (1.9)	1 (0.5)	13 (5.1)	12 (3.7)	13 (4.5)	9 (3.7)	4 (2.1)
**Target vessel related MI**	4 (1.5)	1 (0.5)	12 (4.7)	8 (2.5)	13 (4.5)	7 (2.8)	4 (2.1)
**Target vessel revascularization**	13 (4.9)	5 (2.6)	14 (5.6)	10 (3.1)	14 (4.9)	11 (4.6)	1 (0.5)*
**Target lesion revascularization**	9 (3.4)	4 (2.1)	12 (4.8)	7 (2.2)	9 (3.1)	6 (2.6)	1 (0.5)
**Definite-or-probable stent thrombosis**	2 (0.7)	0	2 (0.8)	1 (0.3)	3 (1.1)	4 (1.7)	0
**Definite stent thrombosis**	2 (0.7)	0	1 (0.4)	1 (0.3)	2 (0.7)	2 (0.8)	0
**Major adverse cardiac events †**	16 (6.0)	7 (3.7)*	32 (12.5)	30 (9.3)	35 (11.9)*	33 (13.2)*	30 (15.6)*

**Target vessel failure ‡**	17 (6.4)	6 (3.2)	26 (10.3)	21 (6.6)	33 (11.4)*	28 (11.3)	17 (9.0)
**Target lesion failure §**	13 (4.9)	5 (2.6)	24 (9.5)*	18 (5.6)	29 (10.0)*	24 (9.7)*	17 (9.0)

Data are n (%), unless otherwise indicated. * p-value of <0.05 † Major adverse cardiac events is a composite of all-cause mortality, any myocardial infarction, emergent coronary artery bypass surgery, and clinically indicated target lesion revascularization. ‡Target vessel failure is a composite of cardiac mortality, target vessel related myocardial infarction, and clinically indicated target vessel revascularization. §Target lesion failure is a composite of cardiac mortality, target vessel related myocardial infarction, and clinically indicated target lesion revascularization.
Abbreviations: MI=myocardial infarction
